# Metformin prevention of doxorubicin resistance in MCF-7 and MDA-MB-231 involves oxidative stress generation and modulation of cell adaptation genes

**DOI:** 10.1038/s41598-019-42357-w

**Published:** 2019-04-10

**Authors:** Poliana Camila Marinello, Carolina Panis, Thamara Nishida Xavier Silva, Renata Binato, Eliana Abdelhay, Juliana Alves Rodrigues, André Luiz Mencalha, Natália Medeiros Dias Lopes, Rodrigo Cabral Luiz, Rubens Cecchini, Alessandra Lourenço Cecchini

**Affiliations:** 1 Laboratory of Molecular Pathology, Department of Pathological Sciences, State University of Londrina, UEL, Londrina, PR Brazil; 2 Laboratory of Tumor Biology, State University of West Parana, Unioeste, Francisco Beltrão, PR Brazil; 3 Stem Cell Laboratory, Bone Marrow Transplantation Unit, National Cancer Institute (INCA), Rio de Janeiro, RJ Brazil; 4 Laboratory of Cancer Biology, Department of Biophysics and Biometrics, State University of Rio de Janeiro, Rio de Janeiro, RJ Brazil; 5 Laboratory of Pathophysiology and Free radicals, Department of Pathological Sciences, State University of Londrina, UEL, Londrina, PR Brazil

## Abstract

Metformin was shown to sensitize multidrug resistant breast cancer cells; however, the mechanisms involved in this capacity need to be clarified. We investigated oxidative stress and inflammatory-related pathways during the induction of doxorubicin resistance in MCF-7 and MDA-MB-231 human breast cancer cells (DOX-res group), and evaluated metformin-induced cellular responses that resulted in the prevention of doxorubicin resistance (Met-DOX group). Microarray analysis demonstrated that DOX-res changed the expression of genes involved in oxidative stress (OS) and the TGF- β1 pathway. The DOX-res group presented increased thiols and reduced lipoperoxidation, increased levels of nitric oxide, nuclear NF-kB and Nrf2, and reduced nuclear p53 labelling. Analysis of the TGF-β1 signaling pathway by RT-PCR array showed that DOX-res developed adaptive responses, such as resistance against apoptosis and OS. Metformin treatment modified gene expression related to OS and the IFN-α signaling pathway. The Met-DOX group was more sensitive to DOX-induced OS, presented lower levels of nitric oxide, nuclear NF-kB and Nrf2, and increased nuclear p53. Analysis of the IFN-α signaling pathway showed that Met-DOX presented more sensitivity to apoptosis and OS. Our findings indicate that metformin is a promising tool in the prevention of chemoresistance in patients with breast cancer submitted to doxorubicin-based treatments.

## Introduction

Breast cancer is a heterogeneous disease. Despite the advances in early diagnosis and targeted therapeutics, an important factor that limits successful treatment is the establishment of resistance to chemotherapy. The mechanisms of chemoresistance are multiple and can be influenced by numerous factors related to individual systemic pharmacology, as well as to single properties acquired by cancer cells, including modifications in cellular pharmacokinetics, leading to drug activation/inactivation, drug-target interactions, altered DNA damage repair mechanisms, evasion of apoptosis and resistance to drug-induced oxidative stress^1^.

The generation of oxidative stress is implied in the mechanism of action of several chemotherapeutic drugs, as evidenced by the systemic detection of altered oxidative profiling in cancer patients after chemotherapy^2^. In this scenario, it is known that some cancer cells develop a chemoresistant phenotype during this pro-oxidant treatment, and may develop additional mechanisms to protect themselves against this chemotherapy-driven oxidative injury^3^.

One of the most used drug to treat solid tumors is doxorubicin^4^. Although it has had relative success in treating breast cancers, studies have described its involvment as a potent inducer of chemoresistance^5^. Considering the importance of chemoresistance in breast cancer therapy, it is imperative to investigate tools to overcome this situation. Metformin has presented antiproliferative effects in numerous breast cancer subtypes *in vitro*^6^, and may sensitize multi-drug resistant breast cancer cells^7^. Qu *et al*.^7^ only investigated the participation of AMPK in this process^7^, but it is known that chemoresistance involves multiple mechanisms, including oxidative stress and inflammation^8^. Therefore, the mechanisms involved in the capacity of metformin to sensitize chemoresistant cells need to be clarified. Furthermore, the interference of metformin in the process of chemoresistance induction has yet to be described.

Considering that understanding the molecular mechanisms used by cells to develop chemoresistance is fundamental to designing efficacious therapies, and to the importance of oxidative stress on the cytotoxic mechanisms of several chemotherapy drugs, in this work, we evaluated certain oxidative stress-related pathways during chemoresistance induction in human estrogen and progesterone receptor positive (MCF-7) and triple negative (MDA-MB-231) breast cancer cells. We also investigated the effects of metformin treatment in the prevention of a doxorubicin-chemoresistant phenotype. The two cell lines were chosen because they correspond to different breast cancer subtypes, with different aggressiveness and treatment responses, MCF-7 were used as corresponding to the luminal subtype, and MDA-MB-231 to the triple negative breast cancer subtype (TNBC); the latter is known to present poor prognosis, due to higher rates of treatment resistance, among other factors. The characterization of the process of chemoresistance induction and metformin effects in the two cell lines were performed in an independent manner.

## Materials and Methods

### Cell culture

MCF-7 cells (*ATCC*^®^ HTB-22™, ATCC, Manassas, VA. USA) and MDA-MB-231 cells (*ATCC*^®^ HTB-26™; ATCC, Manassas, VA, USA) were grown in Dulbecco’s modified Eagle’s medium (Gibco^®^ Life Technologies, Carlsbad, CA. USA) supplemented with 10% fetal bovine serum (DMEM 10% FBS) and 1% penicillin/streptomycin mixture. Cells were maintained in a humidified atmosphere of 5% CO_2_ at 37 °C (Sanyo CO_2_ Incubator; Sanyo, Japan). All experiments were performed in triplicate and repeated three times.

### Induction of doxorubicin resistance

**Figure 1:**
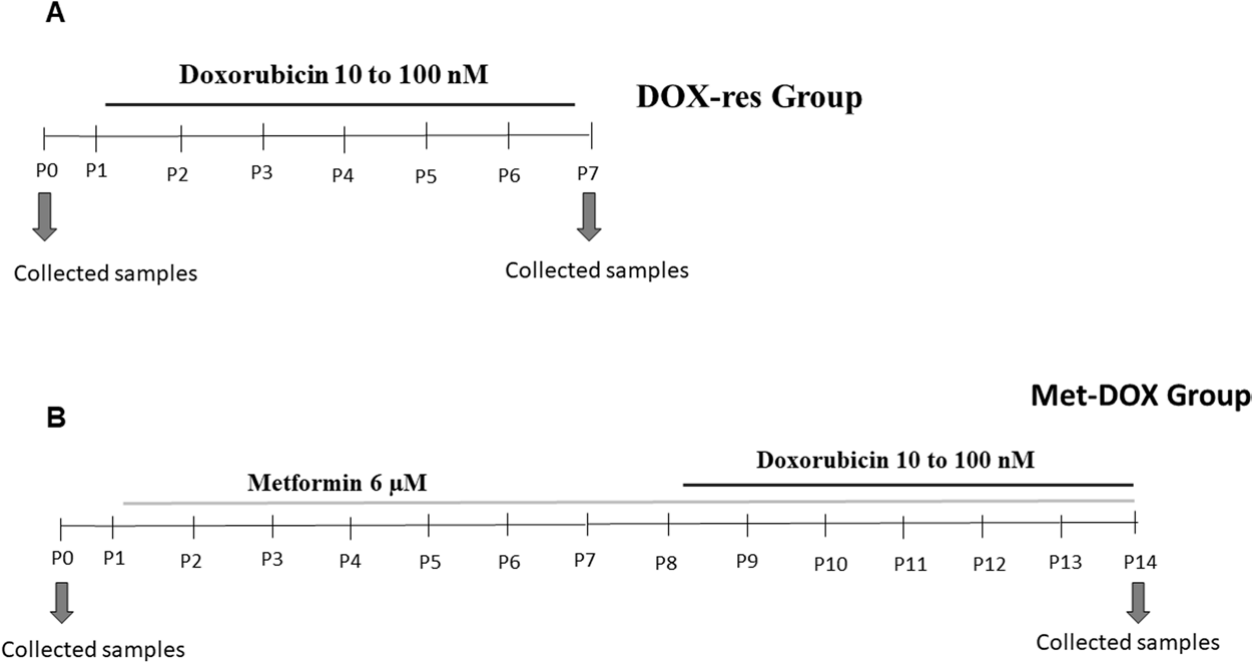
Experimental design for the induction of doxorubicin resistance and metformin treatment. (**A**) Cells were experimentally induced to doxorubicin resistance through the administration of increasing concentrations (10–100 nM) of doxorubicin. DOX-res: Doxorubicin-resistant group (**B**) Cells were pretreated with 6 µM metformin for seven consecutive passages before initiating the process of the induction of doxorubicin resistance. Metformin treatment was maintained during the process of chemoresistance induction. Met-DOX: metformin-treated doxorubicin group.

To induce the doxorubicin-chemoresistant phenotype (DOX-res), MCF-7 and MDA-MB-231 cells were grown in DMEM 10% FBS and exposed to increasing concentrations of doxorubicin (10 nM to 100 nM), as previously described by^9^, during sequential passages. At the beginning of the experiment, 10^6^ cells were seeded in 25-cm^2^ cell culture flasks and after 24 h, the first samples without doxorubicin treatment were obtained; this sample was named as initial passage (P0). Next, cells were trypsinized, counted in a Neubauer chamber and 10^6^ viable cells (determined by trypan blue exclusion assay) were seeded in a new 25-cm^2^ cell culture flask and cultured immediately in culture media with doxorubicin, these samples were also collected and named as P0′. The experimental design for the induction of doxorubicin resistance is illustrated in Fig. 1A. Cells were considered chemoresistant when exposition to increasing concentrations of doxorubicin did not cause their death. In this study, cells became resistant to doxorubicin at the end of seven passages. The samples of the seventh passage were named P7. Throughout the induction period, samples of the initial passage (P0 and P0′) and the final passage (P7) were collected. The same procedures were performed on untreated cells for seven consecutive passages (Control group), to identify possible alterations related to cell passages.

### Metformin treatment

To investigate the effects of metformin on the induction of doxorubicin resistance (Met-DOX), MCF-7 and MDA-MB-231 cells were pretreated with metformin at a non-cytotoxic concentration (6 µM) for seven passages before the induction of doxorubicin resistance. Metformin treatment was maintained during the process of chemoresistance induction. At the beginning of the experiment, 10^6^ cells were seeded in 25-cm^2^ cell culture flasks and after 24 h, we obtained the first samples, in the initial passage (P0), composed of cells without doxorubicin and metformin treatment. Next, cells were trypsinized, counted in a Neubauer chamber by trypan blue exclusion assay and 10^6^ viable cells were seeded in a new 25-cm^2^ cell culture flask and kept in culture media with metformin for 24 h, these samples were also collected and named as P0′. This procedure was repeated for seven consecutive passages. After the seventh passage, the induction of doxorubicin resistance was performed as described above. This group was named metformin-treated doxorubicin (Met-DOX) group and received metformin for fourteen passages and doxorubicin for seven passages. The experimental design for metformin treatment and the induction of doxorubicin resistance is illustrated in Fig. 1B. Throughout the induction period, samples of the initial passage (P0 and P0′) and the final passage of doxorubicin exposure (P14) were collected. A Control group with cells untreated for fourteen consecutive passages was also performed.

### MTT assay

At the end of the process of the induction of doxorubicin resistance, the cells were distributed in 24 well plates (1 × 10^5^ cells/well) and exposed to doxorubicin for 24 h (0.1, 0.5 and 1.0 µM). Then, a MTT assay^10^ was performed to test cell viability. The results were expressed as percentage (%) of control (P7 cells without doxorubicin or metformin treatment) viability.

### Chip array performance

Total RNA from MCF-7 cells from the DOX-res (P0 and P7) and the Met-DOX (P0 and P14) groups were obtained using the RNeasy Mini Kit (Qiagen, CA, USA), according to the manufacturer’s instructions. Total RNA (100 ng) was used to synthesize biotinylated cRNA with the GeneChip^®^ Whole Transcript (WT) Sense Target Labeling Assay (Affymetrix, CA, USA). The biotinylated cRNA was subsequently hybridized to a GeneChip^®^ Human Exon 1.0 ST Array (Affymetrix, CA, USA). Thereafter, the GeneChip^®^ arrays were washed and stained according to the manufacturer’s protocols and scanned using a GeneChip^®^ Scanner 3000. Affymetrix Expression Console software, version 1.0, was used to create summarized expression values (CHP-files). The Robust Multichip Analysis (RMA) algorithm was applied. The data were analyzed using the Partek^®^ software (http://www.partek.com) (Partek^®^, Discovery Suite™. Version 6.3, MO, USA) and a ≥2-fold change in expression was defined as differential overexpression or downregulation in relation to P0 cells.

### Oxidative stress parameters

Total thiol levels^11^ were measured to determine the antioxidant profiling of cells. Lipid peroxidation profile was estimated by chemiluminescence^12^. The results were corrected by number of cells and expressed as µM thiol/10^6^ cells and as area under the chemiluminescence curves (AUC)/10^6^ cells.

### Detection of NO/peroxynitrite profiling by high-sensitivity chemiluminescence

A highly sensitive technique developed by^13^, was modified and used to determine NO levels. The assay was based on the reaction of NO with hydrogen peroxide, which leads to the formation of peroxynitrite. Peroxynitrite in the presence of luminol generates triplet oxygen that decays to singlet oxygen with photon emission. Photon emissions were recorded using a luminometer system and software. Cells were diluted in fresh sterile Na_2_CO_3_ buffer (2 mM; pH: 8.5), which was degassed by N_2_ bubbling for 20 min to eliminate molecular oxygen and prevent the oxidation of NO to nitrite/nitrate. The starting reagent was prepared by mixing equal volumes of luminol solution (4.39 µM dissolved in KOH 1 M) diluted with deferoxamine (36.58 µM) in the ratio 1:10 and H_2_O_2_ (2.44 µM); subsequently, three parts of degassed Na_2_CO_3_ buffer (2 mM; pH: 8.5) was added to the reagent. To the reaction, 500 µL of samples were mixed with an equal volume of buffer. Samples were mixed with 50 µL of the starting reagent and the reaction was performed in a Glomax luminometer (Promega). The results were corrected by number of cells and the peak height was used. Results were expressed as NO peak/10^6^ cells.

### Immunocytochemistry labeling for p53, NF-kB and Nrf2

Immunocytochemistry analysis was performed on coverslip-adherent cells by the labeled streptavidin biotin method using a LSAB kit (DAKO Japan, Kyoto, Japan). The samples were incubated with 0.1% Triton X-100 solution for 1 h, washed 3 times in PBS and treated for 40 min at room temperature with 10% BSA. In addition, coverslips were incubated overnight at 4 °C with the primary antibodies (anti-p53, anti-p65-NF-kB, anti-TGF-β1, diluted 1:400 and anti-Nrf2, diluted 1:300, Santa Cruz Biotechnology). Next, secondary antibody treatment was performed (2 h at room temperature), and horseradish peroxidase activity was visualized by treatment with H_2_O_2_ and 3,3′-diaminobenzidine (DAB) for 5 min. For the final step, the sections were weakly counterstained with Harry’s hematoxylin (Merck). For each case, negative controls were performed by omitting the primary antibody. Intensity and localization of immunoreactivities against the primary antibody used were examined on all coverslips using a photomicroscope (Olympus BX41, Olympus Optical Co., Ltd., Tokyo, Japan). For image analysis, color images of representative areas (400×) were digitally captured. For p53, p65-NF-kB and Nrf2 analyses, the percentage of cells with labeled nuclei were calculated.

### Real-time quantitative array PCR (RT-qPCR)

Analysis of transforming growth factor β1 (TGF-β1) and interferon-alpha (IFN-α) signaling pathway were performed by RT-qPCR. Two micrograms of extracted RNA from MCF-7 and MDA-MB-231 cell lines using RNeasy Mini Kit (Qiagen, CA, USA) were treated with DNAse Amplification Grade I (Invitrogen, CA, USA) and reverse-transcribed with Superscript™ III reverse transcriptase (Invitrogen, CA, USA), according to the manufacturer’s instructions. Diluted cDNAs (1:100) were mixed with SYBR™ Green PCR Master Mix (Applied Biosystems, CA, USA). The TGF-β1 and IFN-α signaling pathway were evaluated using the RT^2^ Profiler™ PCR Array Human TGF-β1 and IFN-α Targets (Qiagen, CA, USA).

### Statistical analysis

Data were expressed as mean ± standard deviation of the mean and analyzed using the paired student t test or one-way ANOVA, followed by Tukey post-hoc. p < 0.05 was considered statistically significant. Data were analyzed using GraphPad Prism (version 6; San Diego, CA, USA).

## Results

### Doxorubicin responsiveness

**Figure 2:**
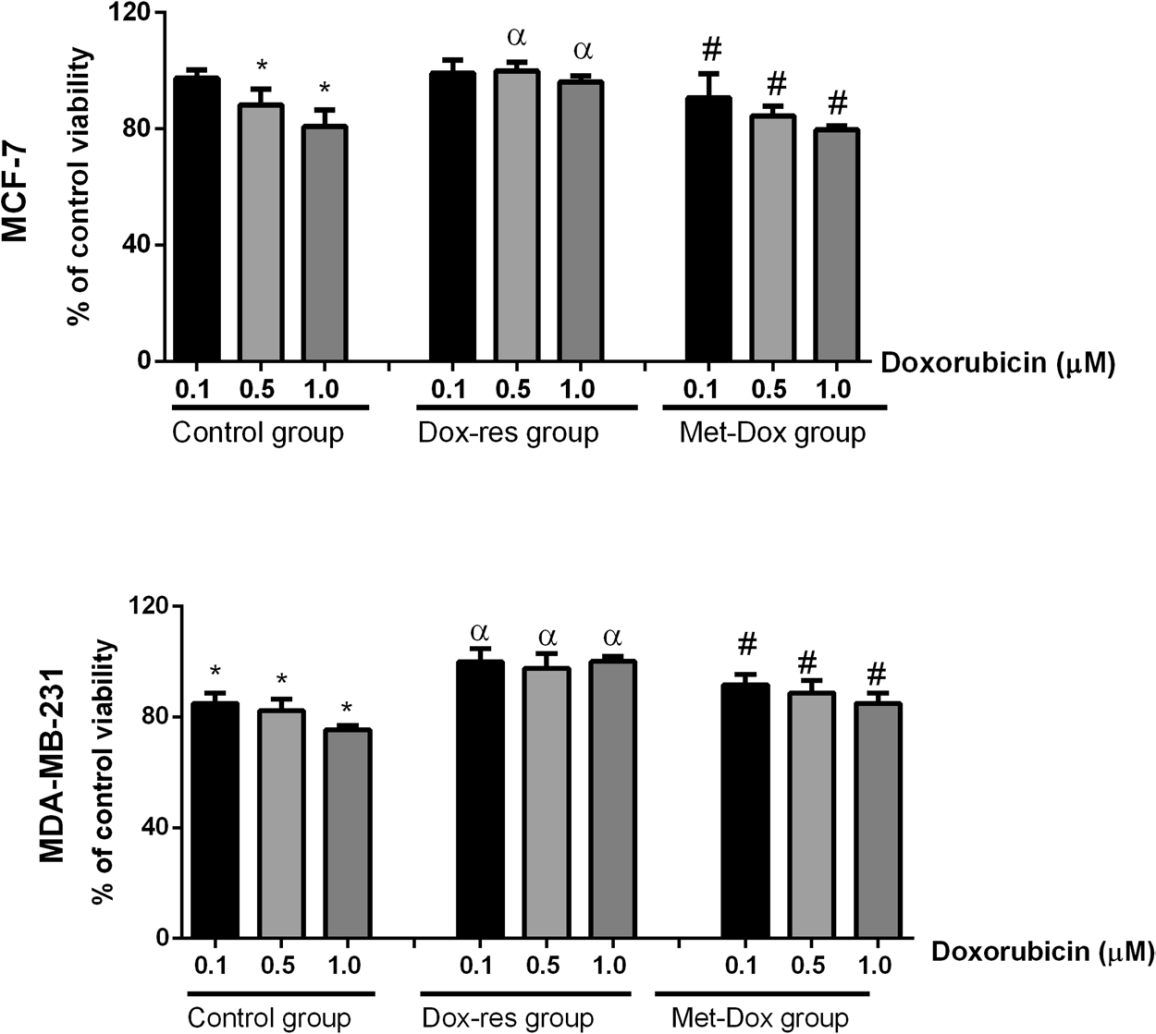
Metformin prevent*s* the induction of doxorubicin resistance. MTT assay of untreated control group, DOX-res and Met-DOX MCF-7 and MDA-MB231 cells after 24 h of exposure to three doxorubicin concentrations (0.1, 0.5 and 1.0 µM). Data are expressed as mean ± standard deviation of mean. Statistically significant differences were investigated by one-way ANOVA, followed by Tukey post-hoc and p < 0.05 was considered significant. The viability of control group was compared to untreated control group (^*^p < 0.05 in this comparison). The different concentrations of doxorubicin in Dox-res group were compared with the same concentration in control group (^α^p < 0.05 in this comparison). The different concentrations of doxorubicin in Met-DOX group were compared with the same concentration in DOX-res group (^#^p < 0.05 in this comparison). DOX-res: Doxorubicin-resistant group. Met-DOX: metformin-treated doxorubicin group.

Figure 2 presents the results of the MTT assay. MCF-7 and MDA-MB-231 control group were sensitive to doxorubicin, since doxorubicin reduced cell viability at the three concentrations tested in MDA-MB-231 and at 0.5 and 1.0 µM in MCF-7 cells. For the highest concentration tested, 1 µM, MCF-7 viability was ±80%, and for MDA-MB-231, it was ±70%. DOX-res groups, for both cell lines, were shown to be extremely resistant with no loss of cell viability. In contrast, metformin treatment significantly prevented the induction of doxorubicin resistance, since the Met-DOX group for both cell lines presented reduced viability.

### Adaptations in doxorubicin-resistant cells

To identify the global gene expression pattern from the DOX-res group, we compared the results of P7 cells with P0 cells for MCF-7. MCF-7 cells were chosen for this analysis, since it has previously been reported that this cell line is more sensitive to doxorubicin than MDA-MB-231 cells^14^, and our results revealed a higher viability for MCF-7 treated with 1 µM doxorubicin. We performed a comparative transcriptome analysis using an expression chip array assay. A ≥ 2-fold change as a cut-off to define overexpression or downregulation, the analysis demonstrated 395 differentially expressed genes in MCF-7 cells after doxorubicin treatment (Supplementary Table 1). Among these, it is possible to highlight the modulation of 74 genes related with oxidative stress, 9 genes related to TGF-β1 signaling pathway and 26 genes related to both oxidative stress and TGF-β1 signaling pathway (Supplementary Table 2). After the alteration in genes related to oxidative stress and TGF-β1, we investigated these pathways in both cells lines.

**Figure 3:**
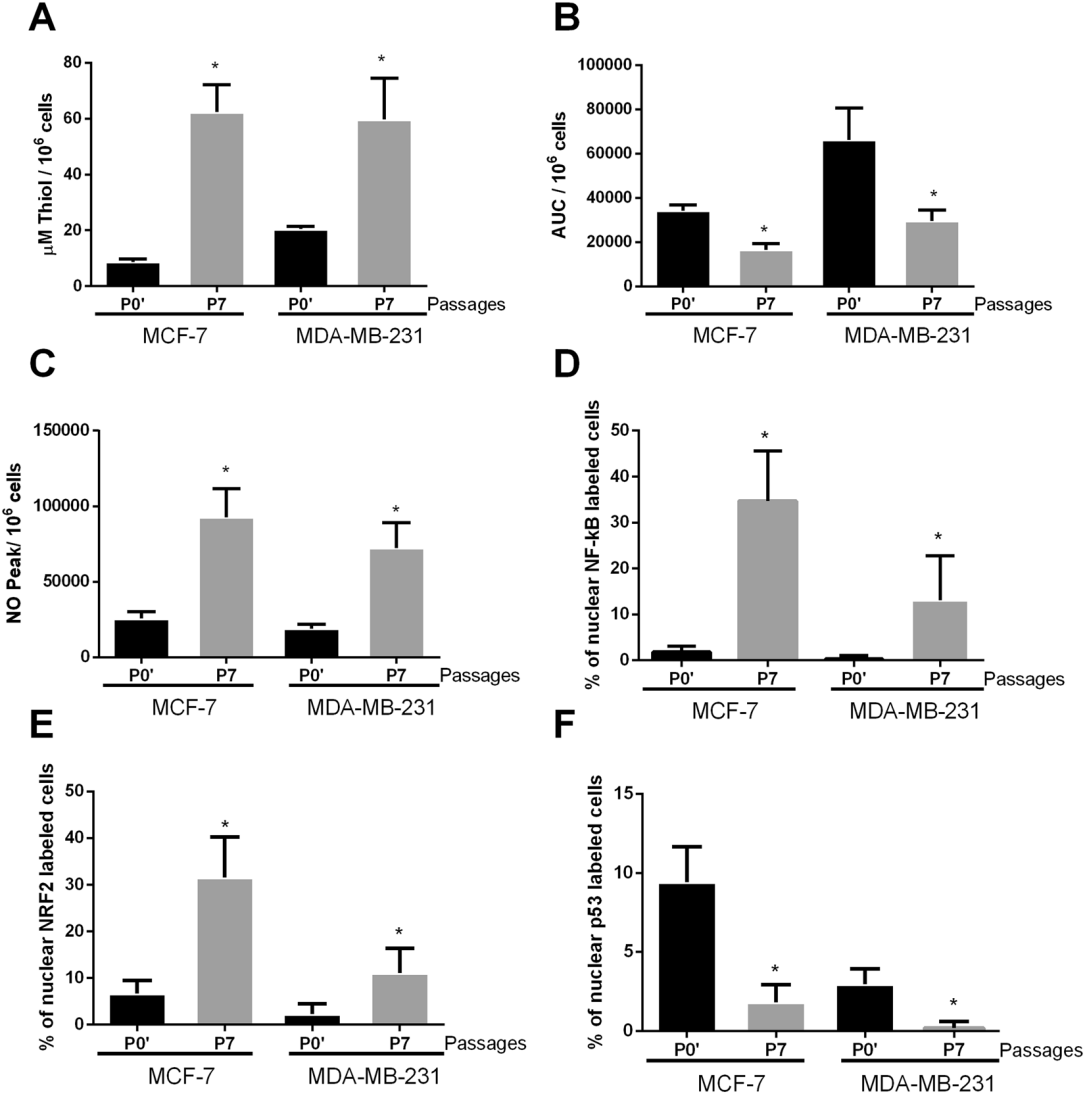
Oxidative profile and related pathways in cells experimentally induced to doxorubicin resistance (DOX-res). (**A**) Total thiol levels, (**B**) area under the chemiluminescence curves and (**C**) peak of the nitric oxide curves in MCF-7 and MDA-MB-231 DOX-res group. (**D–F**) Quantitative results for nuclear p65-NF-kB, Nrf2 and p53, respectively in MCF-7 and MDA-MB-231 DOX-res group. Data are expressed as mean ± standard deviation of mean. Statistically significant differences were investigated by paired student t test and p < 0.05 was considered significant. *Significantly different from the initial passage (P0′).

**Figure 4:**
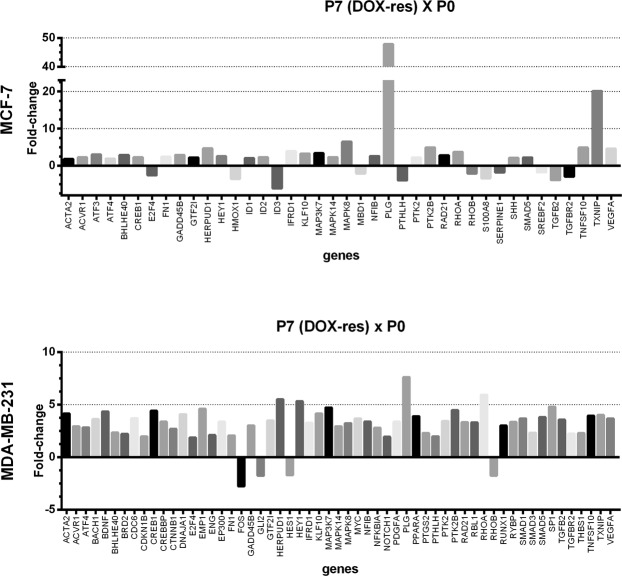
Evaluation of the TGF-β1 signaling pathway in cells experimentally induced to doxorubicin resistance (DOX-res). TGF- β1 signaling pathway related genes showing significantly different expression in MCF-7 and MDA-MB-231 DOX-res group compared with cells in the initial passage (P0), evaluated by RT-PCR array. Data are expressed as fold-change.

The P7 MCF-7 DOX-res group showed increased levels of total thiol in MCF-7 cells (Fig. 3A), reduced lipoperoxidation (Fig. 3B) and increased NO levels (Fig. 3C) when compared to P0′. The cells also presented increased nuclear NF-kB and Nrf2 (Fig. 3D,E, respectively) and reduced nuclear p53 (Fig. 3F and Supplementary Fig. 1). We evaluated 84 genes involved in the TGF-β1 pathway, and among these, doxorubicin treatment altered the expression of 40 genes (Fig. 4). The genes with the most significant variations were PLG (plasminogen), TXNIP (thioredoxin interacting protein), MAPK8 (mitogen-activated protein kinase 8), TNFSF10 (tumor necrosis factor ligand superfamily member 10), VEGFA (vascular endothelial growth factor alpha), PTK2B (protein tyrosine kinase 2 beta), HERPUD1 **(**homocysteine inducible ER protein with ubiquitin like domain 1), RHOA (Ras homolog gene family, member A), all of which were upregulated. Doxorubicin treatment significantly downregulated gene expression of ID3 (inhibitor of DNA binding 3), TGFB2 (transforming growth factor beta 2), TGFBR2 (transforming growth factor beta receptor 2), S100A8 (calcium binding protein A8), PTHLH (parathyroid hormone like hormone), HMOX1 (heme oxygenase 1), E2F4 (E2F transcription factor 4), MBD1 (methyl-CpG binding domain protein 1), SERPINE1 (plasminogen activator inhibitor-1) and SREBF2 (sterol regulatory element binding transcription factor 2).

In MDA-MB-231 cells, the induction of doxorubicin resistance was also accompanied by increased levels of total thiol (Fig. 3A), reduced lipoperoxidation (Fig. 3B) and increased NO levels (Fig. 3C) in P7 compared with P0′. The cells also presented increased nuclear NF-kB and Nrf2 (Fig. 3D,E, respectively) and reduced nuclear p53 (Fig. 3F and Supplementary Fig. 1). In relation to the TGF- β1 pathway, among the 84 genes evaluated, doxorubicin treatment altered the expression of 57 genes (Fig. 4). Some genes were upregulated in both MCF-7 and MDA-MB-231 DOX-res groups, such as PLG, TXNIP, VEGFA, HERPUD1, PTK2B, and RHOA. Alterations were also observed in HEY1 (hes related family bHLH transcription factor with YRPW motif 1), KLF10 (Kruppel like factor 10), TNFSF10 (Tumor necrosis factor ligand superfamily member 10), EMP1 (epithelial membrane protein 1) and CREB1 in MDA-MB-231 DOX-res group, all of which were upregulated. Doxorubicin treatment downregulated the expression of FOS (Fos proto-oncogene, AP-1 transcription factor subunit), GLI2, HES1 (hes family bHLH transcription factor 1) and RHOB.

### Metformin-induced cellular changes involved in reduced doxorubicin resistance

To observe the effects of metformin, we investigated the global gene expression pattern of MCF-7 Met-DOX cells (P14) and compared that with MCF-7 DOX-res cells (P7). A comparative transcriptome analysis using an expression chip array assay was performed. A ≥ 2-fold change was used as a cut-off to define overexpression or downregulation. The analysis demonstrated 138 differentially expressed genes in MCF-7 Met-DOX cells compared with MCF-7 DOX-res cells (Supplementary Table 3). Among these, the modulation of 22 genes related to oxidative stress, 11 genes related to the IFN-α signaling pathway and 2 genes related to both oxidative stress and the IFN-α signaling pathway are worth highlighting (Supplementary Table 4).

**Figure 5:**
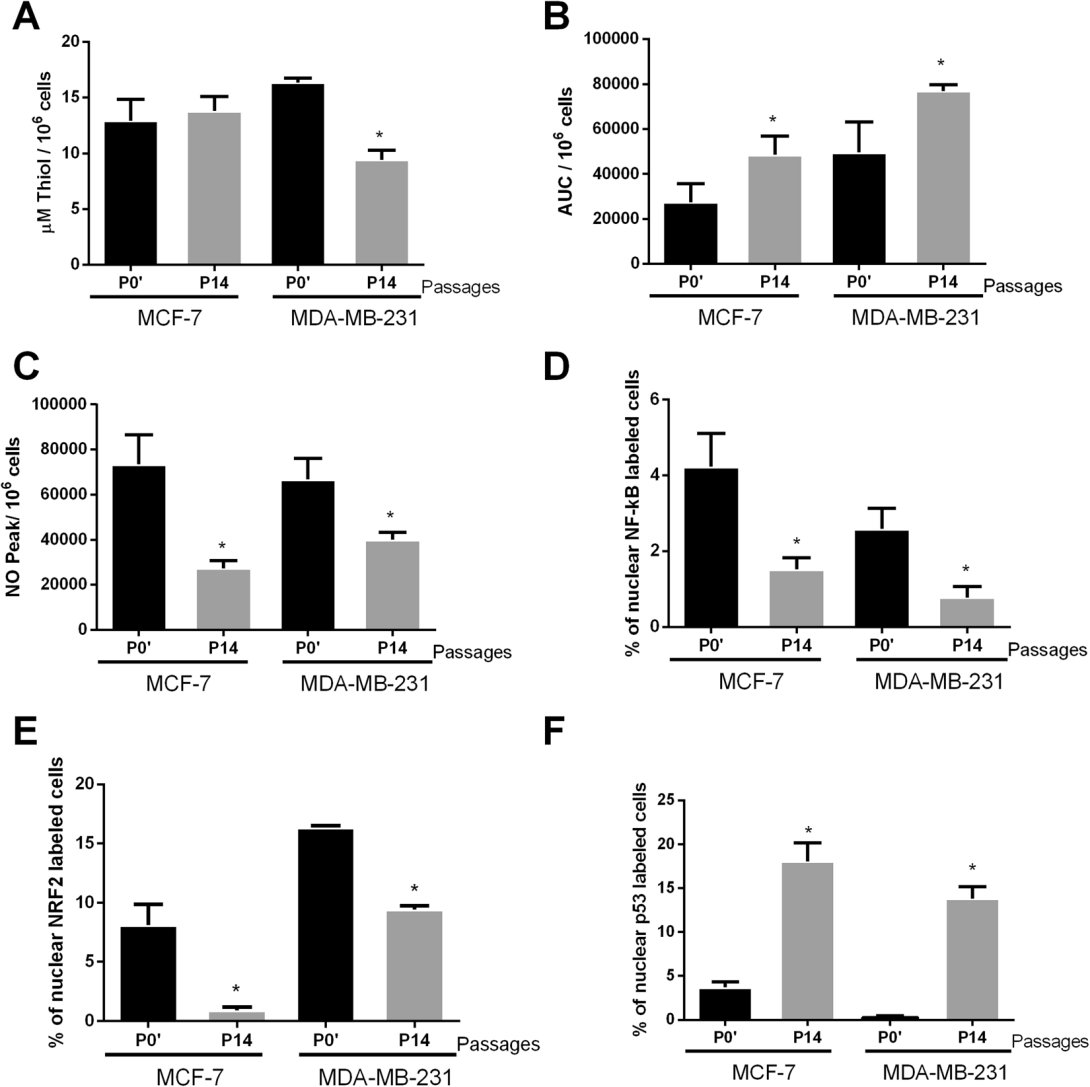
Oxidative profile and related pathways in metformin-pretreated cells experimentally induced to doxorubicin resistance (Met-DOX). (**A**) Total thiol levels, (**B**) area under the chemiluminescence curves and (**C**) peak of the nitric oxide curves in MCF-7 and MDA-MB-231 Met-DOX group. (**D–F**) Quantitative results for nuclear p65-NF-kB, Nrf2 and p53, respectively MCF-7 and MDA-MB-231 Met-DOX group. Data are expressed as mean ± standard deviation of mean. Statistically significant differences were investigated by the paired student t test and p < 0.05 was considered significant. *Significantly different from the initial passage (P0′).

**Figure 6:**
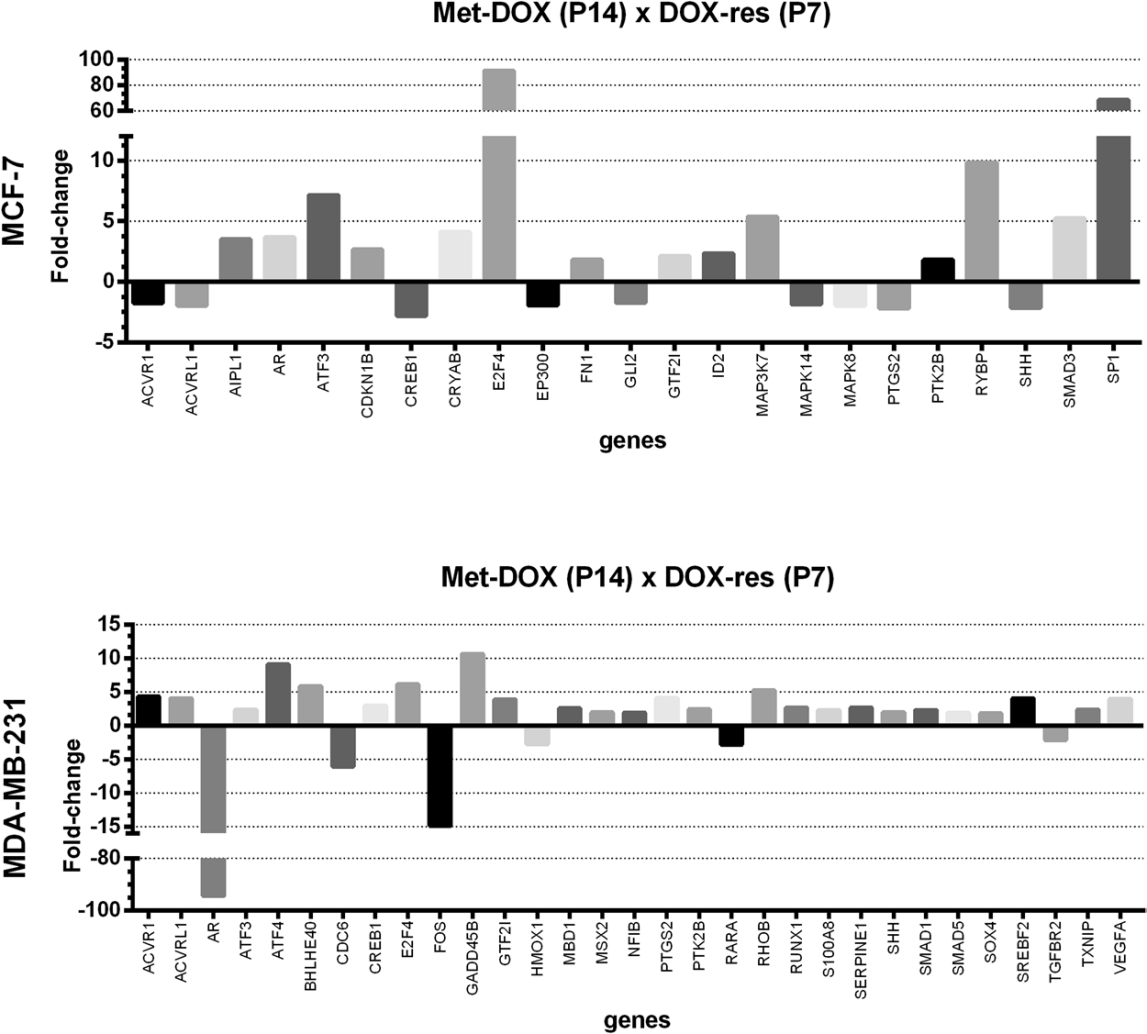
Evaluation of the IFN-α signaling pathway in metformin-pretreated cells experimentally induced to doxorubicin resistance (Met-DOX). IFN-α signaling pathway related genes showing significantly different expression in MCF-7 and MDA-MB-231 Met-DOX group compared with doxorubicin-resistant (DOX-res), evaluated by RT-PCR array. Data are expressed as fold-change.

For the Met-DOX group, we compared the results obtained in the immunocytochemistry and oxidative stress analysis in P14 with P0′. The total thiol levels of MCF-7 Met-DOX cells did not change (Fig. 5A), but lipoperoxidation significantly increased and NO levels decreased (Fig. 5B,C, respectively). Nuclear NF-kB and Nrf2 decreased and nuclear p53 increased in the cells (Fig. 5D–F, respectively). We evaluated 84 genes involved in the IFN-α pathway and among these, P14 MCF-7 Met-DOX cells showed differences in the expression of 23 genes (Fig. 6) compared with P7 MCF-7 DOX-res cells. The genes with the most significant variations were E2F4, SP1(specificity protein 1), RYBP (RING1 and YY1 binding protein), ATF3 (activating transcription factor 3), MAP3K7 (mitogen-activated protein kinase kinase kinase 7), SMAD3 (SMAD family member 3), AIPL1 (aryl hydrocarbon receptor interacting protein like 1), AR (androgen receptor), all of which were upregulated. The Met-DOX-res group presented downregulation of ACVR1 (activin A receptor type 1), ACVRL1 (activin A receptor like type 1), CREB1 (cAMP responsive element binding protein 1), EP300 (E1A binding protein p300), GLI2 (GLI family zinc finger 2), MAPK14 (mitogen-activated protein kinase 14), MAPK8 (mitogen-activated protein kinase 8), PTGS2 (prostaglandin-endoperoxide synthase 2) and SHH (sonic hedgehog).

For MDA-MB-231 cells, the total thiol of P14 decreased in Met-DOX cells (Fig. 5A), lipoperoxidation significantly increased and NO levels decreased (Fig. 5B,C, respectively) compared with P0′. Nuclear NF-kB and Nrf2 decreased and nuclear p53 increased in the cells (Fig. 5D–F, respectively). We evaluated 84 genes involved in the IFN-α pathway and among these P14 MDA-MB-231 Met-DOX cells showed differences in the expression of 31 genes (Fig. 6) compared with P7 MDA-MB-231 DOX-res cells. The MDA-MB-231 Met-DOX cells showed pronounced upregulation in the expression of GADD45B (growth arrest and DNA damage inducible beta), ATF4, E2F4 and BHLHE40 (basic helix-loop-helix family member e40) compared with MDA-MB-231 DOX-res cells (Fig. 6). MDA-MB-231 Met-DOX cells also presented downregulation in the expression of AR, FOS, CDC6 (cell division cycle 6), RARA (retinoic acid receptor alpha) and TGFBR2 compared with MDA-MB-231 DOX-res cells (Fig. 5).

## Discussion and Conclusion

Chemoresistance is one of the main problems that impairs the efficacy of cancer therapy. In this work, we performed an independent characterization of the oxidative stress participation in the process of doxorubicin resistance induction in two cell lines of breast cancer cells. We showed that breast cancer cells develop an adaptive response against the intracellular stress induced by doxorubicin during the process of resistance induction and that a pretreatment of cells with metformin prevented resistance induction.

The experimental process of the induction of doxorubicin resistance, with increasing cellular exposure to the drug, induced the regulation of some intracellular pathways related to cell proliferation and survival, with significant reductions in cellular oxidative stress in MCF-7 and in MDA-MB-231 cells. Besides the reductions in oxidative stress, the chip array results in MCF-7 showed that genes related to both oxidative stress and TGF-β1 were significant modulated in the acquisition of the doxorubicin resistance phenotype. Although other pathways could also be better explored, previous reports relating TGF-β1 levels with treatment resistance (Dai *et al*., 2017) and poor disease prognosis (Panis *et al*.^11^) led us to explore this pathway more thoroughly. Furthermore, an interrelation between TGF-β1 and oxidative stress in cancer has been described (Krstic *et al*., 2015). Immunocytochemistry analysis revealed significant increases in nuclear Nrf2 and NF-kB and reductions in nuclear p53, these proteins were evaluated because of their relation with cell adaptation to environmental stress, like oxidative stress (Pires *et al*., 2018; Kevin *et al*., 2017; Pflaum *et al*., 2014), which could contribute to the chemoresistance-acquired phenotype. The results of Nrf2 analysis indicates that cells respond to the pro-oxidant environment induced by doxorubicin through the activation of Nrf2, a critical transcription factor that regulates antioxidant molecules^15^. Moreover, the basal Nrf2 levels in different cell lines correlate with their respective sensitivity to chemotherapy, specifically in breast cancer cells, low levels of Nrf2 and Nrf2-regulated detoxification proteins, constitute a frequent phenotype in chemosensitive breast cancer cells^1^. Similarly, the increase in Nrf2 levels is associated with both doxorubicin and paclitaxel resistance^1^. The model of the induction of doxorubicin resistance used in this work significantly increased nuclear Nrf2 in the DOX-res group, a result consistent with the significant reduction in cellular oxidative stress, observed by the increase in total thiol levels and the reduction in lipid peroxidation.

The pro-oxidant environment was probably also related to the NF-kB nuclear translocation observed. It is known that the induction of DNA damage by doxorubicin can result in the activation of NF-kB, which could impair drug cytotoxicity^16^. Moreover, doxorubicin activates NF-kB in breast cancer cells and pharmacological inhibition of this pathway increased the antitumor effects of this drug^17^. Clinically, the activation of NF-kB is correlated with resistance to anthracycline-based chemotherapy and worse outcome^5^, suggesting that NF-kB activation during doxorubicin-based treatment is pivotal to the occurrence of poor prognosis events, such as chemoresistance. NF-kB is correlated with the acquisition of chemoresistance^4^ through the induction of anti-apoptotic and pro-survival genes^18^.

The activation of NF-kB could explain the increase in NO levels, since NF-kB can increase the expression of iNOS^19^. NO induces the activation of oncogenic signal transduction pathways (such as c-Myc, Akt and β-catenin) and the loss of PP2A tumor suppressor activity in estrogen receptor-negative breast cancer cells^20^. In addition, NO increases the cellular epithelial mesenchymal transition (EMT), and chemoresistance to doxorubicin and paclitaxel in MCF-7 cells^20^. Our results showed that the augmented NO levels were associated with the development of the chemoresistance, while Nrf2 increase should respond to the attenuation in oxidative stress in the DOX-res group. Doxorubicin resistance was also accompanied by upregulation of VEGFA, which could also be related to increased NO levels. VEGFA activates eNOS and increases the expression of iNOS and eNOS^21^.

The tumor suppressor protein p53 is involved in the regulation of cellular proliferation and apoptosis^22^. Mutations in this protein have been associated with poor clinical prognosis and resistance to chemotherapeutic treatments^23^. Previous research has described that the p53 signaling pathway is necessary to re-sensitize ovarian cancer cells to DNA-damaging chemotherapeutic agents^24,25^. The upregulation in PTK2B that was observed in the DOX-res group could explain the reduction in nuclear p53 observed in the same group, because cytoplasmic tyrosine kinase encoded by this gene limits p53 levels and contributes to cell proliferation and survival^26^. Therefore, the concomitant reduced levels of nuclear p53 and the increased nuclear NF-kB in response to doxorubicin exposure observed in this work in the DOX-res group, corroborate the literature associating the reduction of p53 and increased NF-kB activation with a chemoresistant phenotype.

Evaluation of the genes related to the TGF-β1 signaling pathway confirmed that the DOX-res group developed an adaptive response to protect cells against doxorubicin-induced oxidative stress. Some genes involved in this process were upregulated in both MCF-7 and MDA-MB-231 DOX cells, such as HERPUD1 and RHOA. HERPUD1 is known to exert a cytoprotective role against oxidative stress in cancer cells^27^. Its expression could be induced by Nrf1 and Nrf2 in cells under pro-oxidant conditions in order to block apoptosis through caspase inhibition^27,28^. This modulation probably contributed to the process of cellular adaptation to doxorubicin and chemoresistance. The RHOA pathway signal transduction is also activated by Nrf2^29^ and is related to carcinogenesis, progression, neoangiogenesis and the metastasis of human breast tumors^30^. RHOA is a Ras-GTPase sensitive to oxidative stress that regulates eNOS and iNOS expression to reduce NO levels^31^. Furthermore, both resistant cell lines showed upregulation of PTK2B, which encodes a cytoplasmic protein tyrosine kinase involved in the activation of the MAPK pathway. MCF-7 DOX-res cells presented up regulation of MAPK8 and MDA-MB-231 DOX-res cells presented upregulation of MAP3K7. MAPK8 activation has been previously related to chemoresistance in breast cancer through the reduction of caspase activation and DNA fragmentation after treatment with alkylating agents (mechlorethamine), anthracyclines (doxorubicin), and microtubule inhibitors (paclitaxel)^32^. Similarly, MAP3K7 is a kinase involved in the activation of MAPK8/JNK, MAP2K4/MKK4, and thus plays a role in the cell response to environmental stresses^33^. Both cell lines also presented increases in VEGFA and PLG gene expression. VEGFA has also been previously related to chemoresistance and apoptosis evasion in breast cancer cells^34^ and PLG has been related to highly aggressive characteristics in cancer cells^35^. Besides the alterations in these genes, MCF-7 DOX-res cells also showed downregulation of S100A8, PTHLH and E2F4, all of which have been clinically associated with chemoresistance^36–38^. The evaluation of MDA-MB-231 cells verified upregulation in genes related to highly aggressive characteristics, such as HEY1^39^, EMP1^40^ and CREB1^41^. In this type of cell, the doxorubicin-resistant phenotype was achieved despite the downregulation of genes previously related to chemoresistance, such as FOS^42^, GLI-2^43^, HES1^44^ and RHOB^45^.

Metformin pretreatment significantly prevented the establishment of the doxorubicin-resistant phenotype in both cell lines studied. Our results demonstrated that metformin acted mainly through the induction of cellular oxidative stress and the IFN-α signaling pathway. The Met-DOX group showed a decrease in total thiol levels, increased lipid peroxidation and reduced levels of NO. Moreover, metformin decreased nuclear NF-kB and Nrf2 and increased nuclear p53 in the Met-DOX group (both MCF-7 and MDA-MB-231). When the genes related to the IFN-α signaling pathway were evaluated, metformin was upregulated in both cell lines, together with two genes involved in apoptosis after DNA damage, ATF3 and ATF4^46,47^, compared with the DOX-res group. The upregulation of these two genes is very important to the chemosensitization of cells considering mechanism of action of doxorubicin. Metformin also modulated some genes responsible for the increase in doxorubicin cytotoxicity in MCF-7 cells, such as the upregulation of SMAD3^48^ and AR^49^, and the downregulation of MAPK14, MAPK8^32^ and PTGS2^50^. In MDA-MB-231 Met-DOX cells, metformin upregulated GADD45B, which is a stress-sensor and apoptosis-regulator and contributes to *CDKN1A* upregulation, induction of apoptosis and G2/M-phase enrichment^46^. Furthermore, metformin also upregulated E2F4 in both MCF-7 and MDA-MB-231 Met-DOX cells, which plays a key role in the suppression of proliferation-associated genes^37^. In relation to the effects of metformin on AR^49^, showed that its expression appears to play a different prognostic role in breast cancer positive and negative for estrogen receptors (ER). In ER positive breast cancer, AR was reported to predict favorable disease outcome, its activation directly stimulates the expression of tumor suppressors, like PTEN and KLLN, resulting in p53 activation^49^. In ER negative breast cancer, it is correlated with poor disease outcome^49^. Metformin treatment reduced AR approximately 100-fold in MDA-MB-231 cells exposed to doxorubicin, which corroborates the other beneficial effects promoted by metformin treatment in the Met-DOX group, since the drug upregulated AR in ER + cells (MCF-7) cells and downregulated AR in the ER- cells (MDA-MB-231).

In conclusion, the results of this study propose an oxidative stress-based mechanism for explaining, at least in part, doxorubicin-driven chemoresistance. Furthermore, our data suggest that, although the two cell lines presented some particularities of response, in general metformin generates more oxidative stress, preventing cells from adapting to doxorubicin treatment. These findings indicate metformin as a putative candidate for future trials regarding the prevention or the reversal of acquired chemoresistance in patients submitted to doxorubicin-based treatment^51–56^.

## Supplementary information

Supplementary information

Supplementary Table 1

Supplementary Table 2

Supplementary Table 3

Supplementary Table 4
