# Supplementary material for: Metformin prevention of doxorubicin resistance in MCF-7 and MDA-MB-231 involves oxidative stress generation and modulation of cell adaptation genes
Source: Sci Rep. 2019 Apr 10;9:5864. doi: 10.1038/s41598-019-42357-w (PMC6458149; doi:10.1038/s41598-019-42357-w)
Supplement: Supplementary file 1 — Supplementary information [file 41598_2019_42357_MOESM1_ESM.pdf]

Metformin prevention of doxorubicin resistance in MCF-7 and MDA-MB-231 involves oxidative stress generation and modulation of cell adaptation genes

Poliana Camila Marinello, Carolina Panis, Thamara Nishida Xavier Silva, Renata Binato, Eliana Abdelhay, Juliana Alves Rodrigues, André Luiz Mencalha, Natália Medeiros Dias Lopes, Rodrigo Cabral Luiz, Rubens Cecchini, Alessandra Lourenço Cecchini

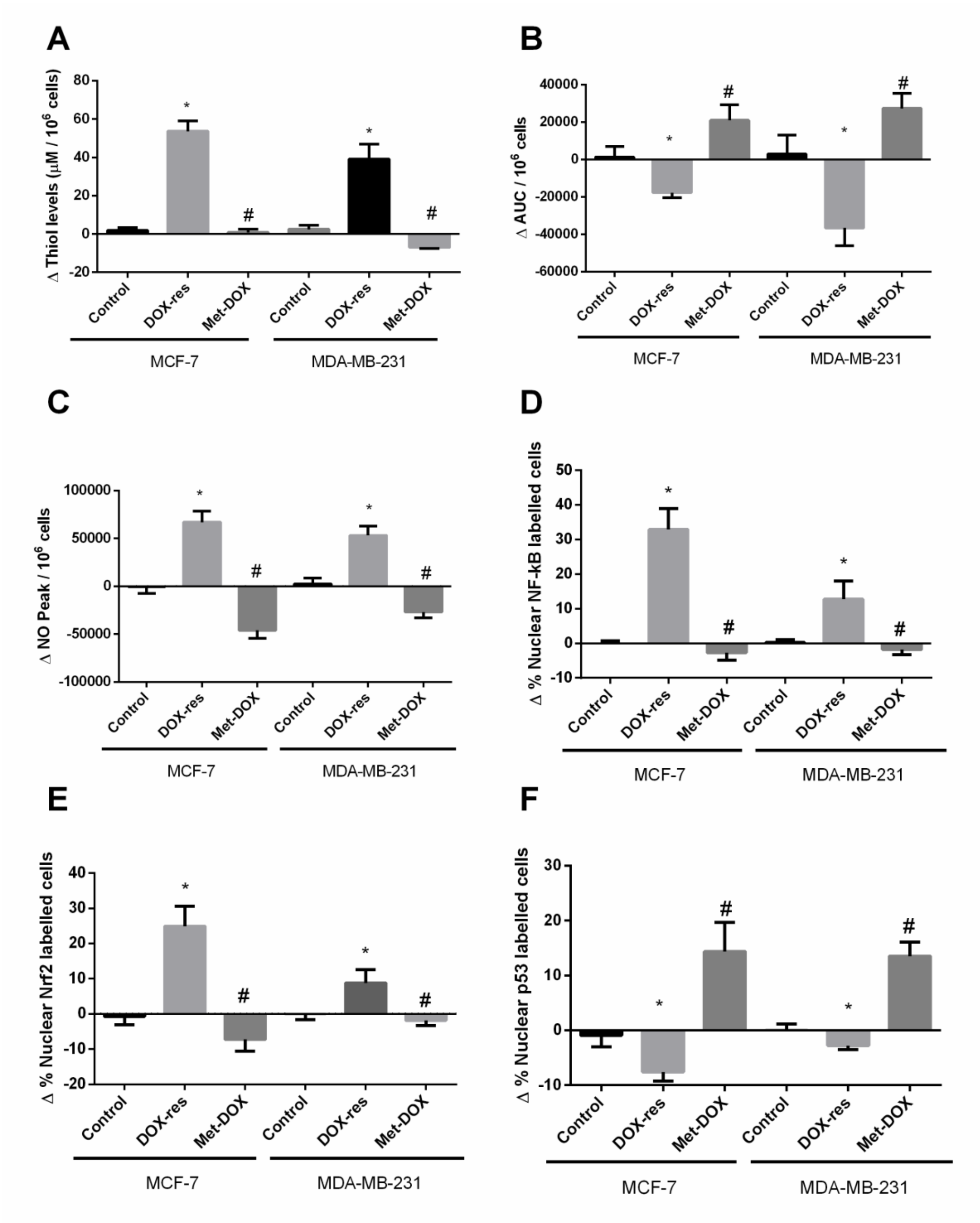

Supplementary figure 1. Variations in the oxidative profile and related pathways in the control group, doxorubicin resistant group (DOX-res) and in metformin-pretreated cells experimentally induced to doxorubicin resistance (Met-DOX). (A) Total thiol levels, (B) area under the chemiluminescence curves and (C) peak of the nitric oxide curves in MCF-7 and MDA-MB-231 cells. (D, E and F) Variations in the quantitative results for nuclear p65-NF-kB, Nrf2 and p53, respectively, in MCF-7 and MDA-MB-231 cells. Data are expressed as mean ± standard deviation. Statistically significant differences were investigated by one-way analysis of variance (ANOVA), followed by Tukey post-hoc and $p<0.05$ was considered significant. The Dox-res group was compared with the control group (*: $p<0.05$ in this comparison). The Met-DOX group was compared with the DOX-res group (#: $p<0.05$ in this comparison).

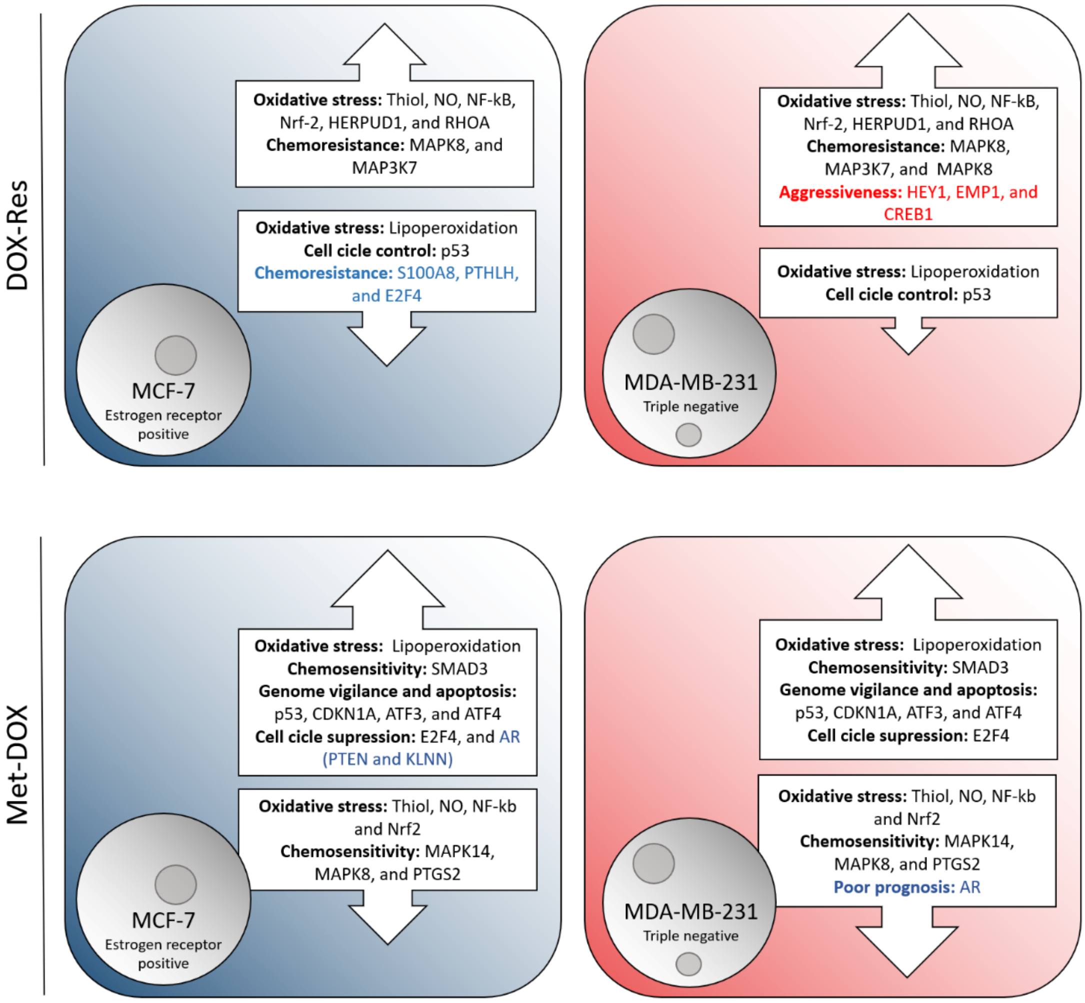

Supplementary figure 2. Summary of the main findings regarding the modulation of oxidative stress and related pathways in doxorubicin resistance and in metformin-pretreated cells experimentally induced to doxorubicin resistance (Met-DOX) in MCF-7 and MDA-MB-231 cells.
